# Pharmacological activation of the amygdala, but not single prolonged footshock-induced acute stress, interferes with cue-induced motivation toward food rewards in rats

**DOI:** 10.3389/fnbeh.2023.1252868

**Published:** 2023-09-14

**Authors:** Chien-Wen Lai, Chun-hui Chang

**Affiliations:** ^1^Institute of Molecular Medicine, National Tsing Hua University, Hsinchu, Taiwan; ^2^Institute of Systems Neuroscience, National Tsing Hua University, Hsinchu, Taiwan; ^3^Brain Research Center, National Tsing Hua University, Hsinchu, Taiwan

**Keywords:** basolateral nucleus of the amygdala, central nucleus of the amygdala, acute stress, motivated behavior, food-seeking

## Abstract

In the face of threats, animals adapt their behaviors to cope with the situation. Under such circumstances, irrelevant behaviors are usually suppressed. In this study, we examined whether food-seeking motivation would decrease under activation of the amygdala, an important nucleus in the regulation of stress response in the central nervous system, or after a physical acute stress session. In Experiment 1, we pharmacologically activated the basolateral nucleus (BLA) or the central nucleus of the amygdala (CeA) before a cue-induced reinstatement test in rats. Our results showed that activation of the BLA or the CeA abolished cue-induced motivation toward food rewards, while locomotor activity and free food intake were not affected. In Experiments 2 and 3, we further assessed anxiety and despair levels, as well as cue-induced reinstatement, after a single prolonged footshock-induced acute stress in rats. Behaviorally, acute stress did not affect anxiety level, despair level, or cue-induced motivation toward food rewards. Physiologically, there was no difference in cellular activities of the amygdala immediately after acute stress. To conclude, our results suggested that pharmacological activation of the amygdala decreased cue-induced motivation toward food reward. However, physiological acute stress did not immediately interfere with the negative emotions, motivation, or amygdala activities of the animals.

## 1. Introduction

In the face of threats, it is vital for individuals to generate appropriate responses to enhance their chances of survival. As stated in the predatory imminence theory, animals adjust their behaviors according to the potential of threats from predators (Fanselow and Lester, 1988). For example, when predators are likely to appear, the animals stay alert (pre-encounter defensive behavior). However, when predators are already in direct contact, the animals elicit “*circa*-strike behavior” to escape. In order to adjust to different scenarios, specific neuronal systems are recruited to guide animal behavior (Duval et al., 2015; Meyer et al., 2021), among which the amygdala acts as one of the brain regions involved in such regulation (Davis et al., 1994; Öhman, 2005; Roozendaal et al., 2007, 2009). For example, the basolateral nucleus of the amygdala (BLA), which is one of the important nuclei to regulate emotions, is activated under acute stress (Cullinan et al., 1995; Kavushansky and Richter-Levin, 2006; Reznikov et al., 2008). The central nucleus of the amygdala (CeA) receives robust projection from the BLA (Pitkänen et al., 1995; Savander et al., 1995) and projects to diverse downstream targets in the regulation of behavioral and physiological responses (Janak and Tye, 2015; Zhang et al., 2021), is also activated under acute stress (Dayas et al., 2001; Reznikov et al., 2007). In a recent study, it was shown that the animals shifted their defensive behavioral strategies based on how they perceived the imminence of the threats in a series of behavioral assays (Hoffman et al., 2022). These results led to the likelihood that acute stress prepared the animals to cope with the challenge by adjusting their behavioral strategies instead of merely leading to typical anxiety- or fear-related behaviors. These results also raised another question: that when defensive behaviors were engaged, whether the reward system became suppressed under such circumstances.

In human studies, subjects showed higher amygdala activities during the anticipatory phase of reward processing under acute stress (Kumar et al., 2014). However, acute stress in laboratory settings reduces reward responsiveness, learning, and sensitivity (Bogdan and Pizzagalli, 2006; Morris and Rottenberg, 2015; Burani et al., 2021). In our previous study, animals displayed different anxiety levels based on different task demands under pharmacological activation of the BLA, and there was a down-regulation of dopamine (DA) population activity in the ventral tegmental area (VTA) in the meantime (Lai and Chang, 2019). The DA population activity, defined as the number of spontaneously firing DA neurons, plays a role in the reward system and determines the magnitude of behavioral response (Grace et al., 2007; Arias-Carrián et al., 2010). Although DA activity increased shortly after acute stress, it subsequently decreased after 18 h in rodent studies (Puglisi-Allegra et al., 1991; Valenti et al., 2011; Chang and Grace, 2013; Belujon et al., 2015). As a result, down-regulation of the DA reward system under BLA activation or after acute stress may lead to a decrease in the motivation of the animals. Indeed, researches have shown that DA depletion in the nucleus accumbens (NAc) decreased the effort-demanded response for food rewards (Aberman and Salamone, 1999; Salamone et al., 2001).

The physiological and behavioral response to acute stress is mediated through a complex system, such as activation of the hypothalamic–pituitary–adrenal (HPA) axis (Johnson et al., 1992; Kudielka and Kirschbaum, 2004) and sympathetic division of autonomic nervous system (ANS) (McCorry, 2007). Several brain nuclei of the central nervous system are also involved in stress regulation, such as increased activities in the amygdala (McEwen, 2007; Dedovic et al., 2009). In this study, we focused on how amygdala activation or single prolonged physical acute stress-regulated motivation. We predicted that the appetitive motivation of the animals would be suppressed under these circumstances. Several behavioral tasks can be used to evaluate motivation, such as outcome devaluation tasks, effort-based tasks, and reward-seeking (Markou et al., 2013). In effort-based tasks, the animals are trained with a fixed ratio schedule (Aberman and Salamone, 1999; Salamone et al., 2001; Simmons and Neill, 2009; De Jong et al., 2015) or a progressive ratio schedule (Zhang et al., 2003; De Jong et al., 2015; Randall et al., 2015). These schedules are used to assess how much effort the animals are willing to pay to get the rewards. On the other hand, reinstatement tests are used to examine the motivation of drug-or food-seeking (Ahmed et al., 2000; Yager and Robinson, 2010; Moorman et al., 2017). We are especially interested in cue-induced motivation toward food rewards in reinstatement tests. During instrumental training, the reward-associated cue becomes a feature of incentive stimulus and the incentive salience is attributed to the DA system (Pitchers et al., 2018). In Experiment 1, the rats were tested under pharmacological activation of the BLA or the CeA, the latter serving as the major output nucleus of the amygdaloid complex. We hypothesized that BLA or CeA activation would decrease the cue-induced motivation toward food rewards in reinstatement tests. In Experiment 2, the rats were tested for behavioral assays after a single prolonged footshock-induced acute stress session, to examine if the acute stress affected anxiety and despair level. We hypothesized that footshock-induced acute stress would increase anxiety and despair levels. In Experiment 3, the rats were tested with cue-induced reinstatement after acute stress and cellular activities were assessed with an immediate early gene of c-fos expression in the amygdala, the NAc core (NAcc), and the VTA DA neurons. We hypothesized that footshock-induced acute stress would decrease the cue-induced motivation toward food reward in reinstatement tests accompanied by increased cellular activities in the amygdala. We also hypothesized that reinstatement tests would increase cellular activities in the NAcc and the VTA DA neurons, but the level of increase would be lower following acute stress.

## 2. Experimental procedures

### 2.1. Subjects

A total of 90 male Long-Evans rats (250–300 g; National Laboratory Animal Center, Taiwan) were used in the study. The rats were individually housed in a temperature- (21–22°C) and humidity- (60–70%) controlled facility on a 12 h light/dark cycle (lights on at 7:00 a.m.) with food and water available *ad libitum*. The rats were handled for at least 5 days for 10 s/day before any experimental procedures. All procedures were performed according to the protocols approved by Institutional Animal Care and Use Committees (IACUC) of both National Tsing Hua University and National Yang Ming Chiao Tung University.

### 2.2. Surgery

In Experiment 1, the rats were anesthetized with ketamine (80–100 mg/kg, i.p.) and xylazine (10 mg/kg, i.p.), and then were placed on a stereotaxic apparatus (Stoelting). The half-life of ketamine is about 1.5 h (White et al., 1975; Veilleux-Lemieux et al., 2013). The core body temperature was maintained at 37°C by a temperature-controlled heating pad (CWE). Burr holes were drilled above bilateral BLA [from bregma: anterior–posterior (AP) -2.8 mm; mediolateral (ML) +5.2 mm; dorsoventral (DV) −7.6 mm] or CeA [from bregma: (AP) −2.1 mm; (ML) +4.0 mm; (DV) −7.2 mm]. Two 26-gauge stainless steel guide cannulae (Plastics One) were implanted to target the BLA or the CeA. Three anchor screws were mounted on the skull and the headstage was covered with dental acrylic. The rats were injected with carprofen (5 mg/kg, s.c.) for analgesic. At least 5 days of recovery were allowed before drug administration or behavioral assays, while dummy cannulae (Plastics One) extended 1.0 mm beyond the end of the guide cannulae were changed daily.

### 2.3. Drug administration

In Experiment 1, drugs were given immediately before behavioral tests. The drugs were delivered through injectors (33-gauge, Plastic One) protruding 1.0 mm past the end of the guide cannulae. The injectors were attached to polyethylene tubes and connected to Hamilton syringes (5.0 μL) located on a micro-infusion pump (Harvard Apparatus). The rats were infused with N-methyl-D-aspartate (NMDA, 0.05 μg in 0.5 μL/side) or saline as a control. The dosage was chosen based on previous studies (Rezayof et al., 2007; Solati and Hajikhani, 2010) and was much lower than the concentration used for the lesion procedure (Sananes and Davis, 1992; Roozendaal and McGaugh, 1997; Zimmerman et al., 2007). Previous electrophysiology studies using higher (Legault et al., 2000; Floresco et al., 2001) or lower (Pisani et al., 1997; Shen et al., 2007; Zhang et al., 2022) NMDA concentration in comparison to ours have demonstrated physiological effects under their selective dosages. The drugs were delivered at a rate of 0.2 μL/min for 2 min and 30 s. An additional minute was allowed for drug diffusion before the injectors were removed. The series of drug delivery behavioral tests were counterbalanced. Some subjects were excluded from analyses after the experimental procedure due to histology check, and therefore the group size may not be equally distributed in every drug delivery condition.

### 2.4. Single prolonged stress paradigm

In Experiments 2 and 3, a single prolonged footshock-induced acute stress was conducted before the reinstatement test. The procedure was conducted in sound-attenuating fear conditioning cubicles (Med-Associates). To generate a setting different from the operant chambers, the ceiling lights and the fans attached to the cubicles remained off, while A-frames were inserted. To provide a distinct odor, stainless-steel pans containing a thin layer of 1% ammonium were placed underneath the grid floors before the rats were placed inside. The same solution was used to clean the chambers between squads. The procedure was adapted from the Arakawa study (Arakawa et al., 2014). During the 2 h session, 5-s footshocks (1 mA) were delivered 80 times. The intertrial interval (ITI) was 90 s on average (70–110 s). The video was recorded in Experiment 3 and the freezing behavior was analyzed with Video Freeze (Med-Associates). The freezing behavior was defined as consecutive observed movements below the motion threshold (program setting at 18) for 1 s (video frame sampling at 0.2 s; e.g. at least continuous six frames below threshold). The “Stress” group of animals underwent the above procedure immediately before the behavioral test, while the “Ctrl” group of animals stayed in their home cages.

### 2.5. Behavior

#### 2.5.1. Cue-induced reinstatement test

The experiment was conducted in sound-attenuating operant chambers (Med Associates). There were two retractable levers equipped on one of the chamber walls, with stimulus lights above the levers and a food port in between. During behavioral training and tests, the chamber lights and the fans attached to the chambers remained on. To provide a distinct odor, stainless-steel pans containing a thin layer of 1% acetate acid were placed underneath the grid floors before the rats were placed inside. The same solution was used to clean the chambers between squads. The procedure was adapted from the study by McLaughlin and Floresco (2007). Before training, the animals were food deprived to 85–90% of their free-feeding weight. It typically took 5 days to reach this criterion. The food deprivation process continued to the end of all experiments and the rats were weighed daily to ensure they maintained between 85 and 90% of their free-feeding weight. The first 2 days consisted of 30-min behavioral shaping sessions, where reward food pellets (45-mg sucrose pellets, Bio-serv) were dispensed on a fixed-ratio (FR) 1 schedule of reinforcement. On the following days, animals received 20-min lever pressing reinforcement training sessions. During training, both levers were introduced with the left lever designated as the active lever and the right lever as the inactive lever. On the first day of training, the reinforcement schedule was set to a FR1 schedule. Delivery of a food pellet was always paired and preceded by a 5 s light/tone conditioned stimulus (CS), which entailed illumination of the stimulus light above the active lever and presentation of an 80 dB, 3 k Hz tone. This was followed by a 20-s time-out period, where pressing the levers did not result in food pellet/CS delivery. On the second day, the schedule was increased to an FR2 schedule without a 20-s time-out period. A variable-ratio (VR) 5 schedule was implemented on the following 5 days, ensuring that the animals responded reliably to the active lever by the end of training. The rats then underwent daily 20-min extinction sessions, where neither food nor the light/tone CS were presented after responding with either lever. Extinction sessions continued on subsequent days until a cohort of animals reached the criterion, in which the mean number of responses on the active lever was lower than 10% of presses relative to the last day of the VR5 schedule. The animals typically took four to 6 days to reach this criterion. On the day after the extinction criterion was reached, animals were subjected to the 20-min reinstatement test, where the first CS was presented 5 s after the session started. During the test session, pressing the active lever elicited the presentation of the light/tone CS in the absence of food pellets on a VR5 schedule. The lever pressing number was recorded in every session. In Experiment 1, all the animals underwent the reinstatement test procedure. In Experiment 3, the “Rein” group of animals underwent the reinstatement test procedure, while the “No Rein” group of animals stayed in their home cages. The following two parameters were additionally analyzed in Experiment 3. (a) Nosepoke was defined as whenever the rats placed their nose into the food port. (b) Nosepoke latency was defined as the delay from the start of CS to the first nosepoke. If the rat did not nosepoke after the presence of CS, it was defined as an “omission” and was not included in the analysis of latency.

#### 2.5.2. Locomotor

Rats were placed in an open-field arena (Med associates) for 30 min. The total traveling distance (cm) was measured.

#### 2.5.3. Free-feeding

Rats were placed in a 45 × 21 × 20 cm^3^ cage box with unlimited food pellets (45 mg sucrose pellets, a total of 140–200 g) for 30 min. The consumed food pellets (g) were calculated.

#### 2.5.4. Elevated plus maze

One day before the test, the rats were placed in the behavioral room in their home cages to habituate to the environment for 2 h. On the test day, each rat was placed in the center of the maze facing one of the closed arms, with video recording for 5 min. (a) Time spent in the open arms as the percentage of time spent in open and closed arms, and (b) entries into the open arms as the percentage of entries into open and closed arms, were calculated.

#### 2.5.5. Forced swim test

The elevated plus maze (EPM) and forced swim test (FST) were conducted on the same days. For FST, the pre-exposure and test were performed in a cylinder (50 cm in height × 20 cm in diameter) filled with water (23–25°C) to 30 cm. After the habituation procedure of EPM, each rat underwent the pre-exposure procedure of FST. The rats were individually placed in the cylinder for 15 min and this procedure was to ensure that they would quickly adapt to immobility on the test day. On the next day, after being tested with EPM, each rat was placed in the cylinder for 5 min for FST with video recording. The time spent in immobility was calculated.

### 2.6. Histology

**Figure 1:**
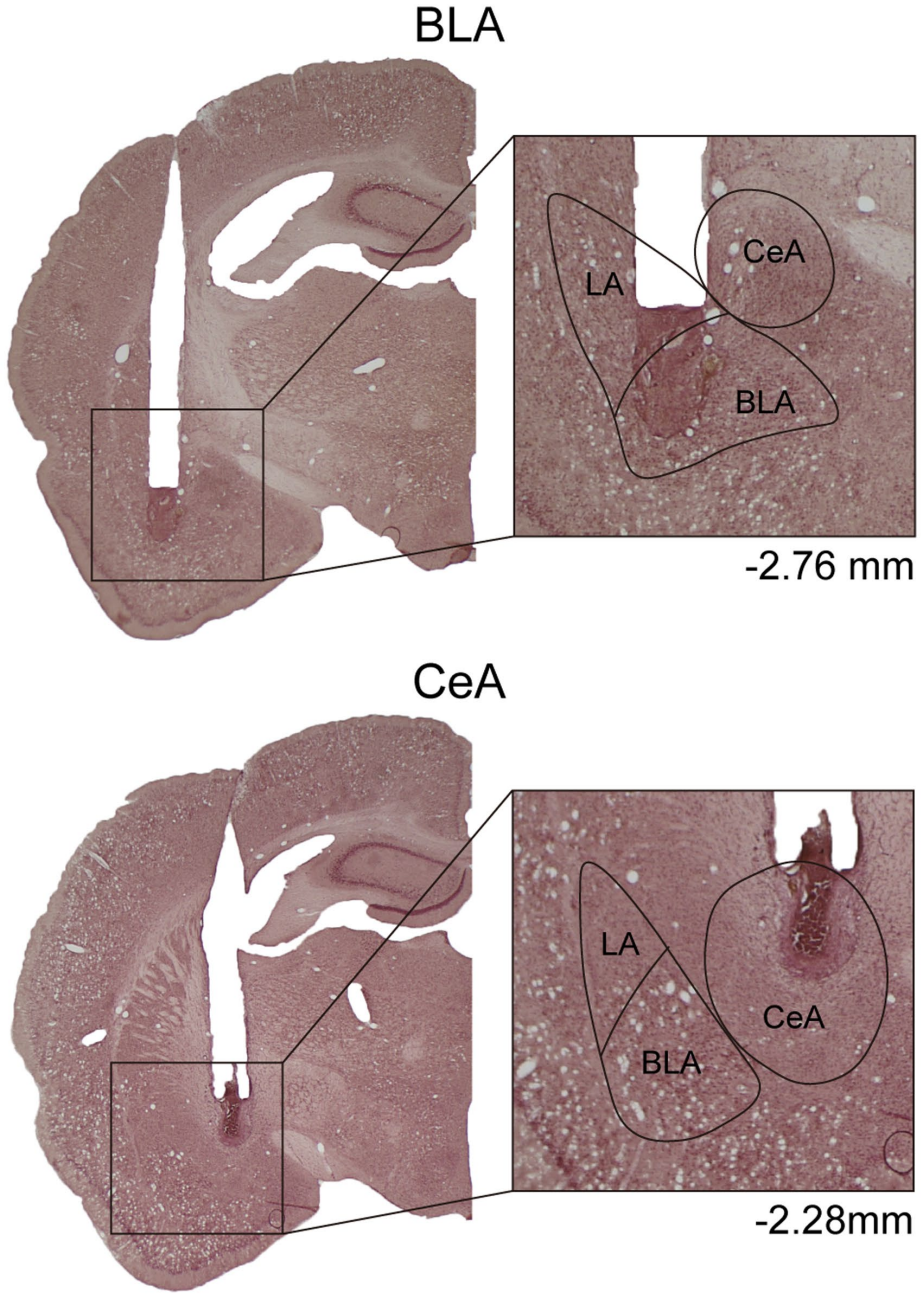
Example of cannula placements in the BLA and the CeA in Experiment 1. The location of injector tips on the nissl-stained brain sections was defined as the drug infusion site. LA, lateral nucleus of the amygdala; BLA, basolateral nucleus of the amygdala; CeA, central nucleus of the amygdala.

In Experiment 1, to verify the cannula placements, the rats were deeply anesthetized with CO_2_, decapitated, and then their brains were removed. The brains were post-fixed at least 48 h in 8% paraformaldehyde (PFA) in 0.2 M phosphate buffer (PB) and cryoprotected with 25% sucrose in 0.1 M PB until saturated. The brains were sectioned coronally by 35 μm and collected sequentially into six adjacent sets and stored in cryoprotectant at −20°C. One tissue section series (containing sections spaced by 210 μm) from each rat was mounted onto gelatin-coated slides and stained with nissl solution (a combination of neutral red and cresyl violet) for histological verification of drug infusion sites (Figure 1).

In Experiment 3, to capture the maximum expression of c-fos, the rats were sacrificed 90 min after the behavioral test. The “No Rein” group was sacrificed along with the “Rein” group. The rats were deeply anesthetized with CO_2_ and perfused transcardially with 50 mL of saline followed by 100 mL of 4% PFA in 0.1 M PB. Brains were removed and post-fixed for at least 4 hours and then cryoprotected in 25% sucrose in 0.1 M PB. The brains were sectioned coronally by 35 μm and collected sequentially into six adjacent sets and stored in cryoprotectant at −20°C. One tissue section series (containing sections spaced by 210 μm) from each rat was mounted onto gelatin-coated slides and stained with nissl solution. Another section series was used for later immunohistochemical processing.

### 2.7. Immunohistochemistry

In Experiment 3, the c-fos and tyrosine hydroxylase (TH) visualization were performed with standard immunohistochemical procedures. Free-floating brain sections were incubated overnight in phosphate buffer saline solution (PBS) with mouse anti-c-fos antibody (Santa Cruz, catalog#: sc-271243, 1:200). On the following day, after washing with buffer, the sections were incubated in biotinylated donkey anti-mouse IgG solution (Jackson Immuno Research, catalog#: 715–065-151, 1:500), and then Vectastatin ABC Elite reagents (Vector Laboratories), followed by a solution of nickel sulfate and diaminobenzidine (DAB) with hydrogen peroxide to produce a blue-black reaction product within the nucleus of c-fos + neurons. The sections were then incubated overnight in buffer solution with rabbit anti-TH antibody (Merck Millipore; Catalog#: 6A2907, 1:5,000). On the following day, after washing with buffer, the sections were incubated in biotinylated donkey anti-rabbit IgG solution (Jackson Immuno Research, catalog#: 711–065-152, 1:500), and then Vectastain ABC Elite reagents, followed by a solution of non-intensified DAB with hydrogen peroxide to produce a brown reaction product within the cytosol of TH+ neurons. The sections were then rinsed in the buffer and mounted onto gelatin-coated slides.

### 2.8. Image analysis

**Figure 2:**
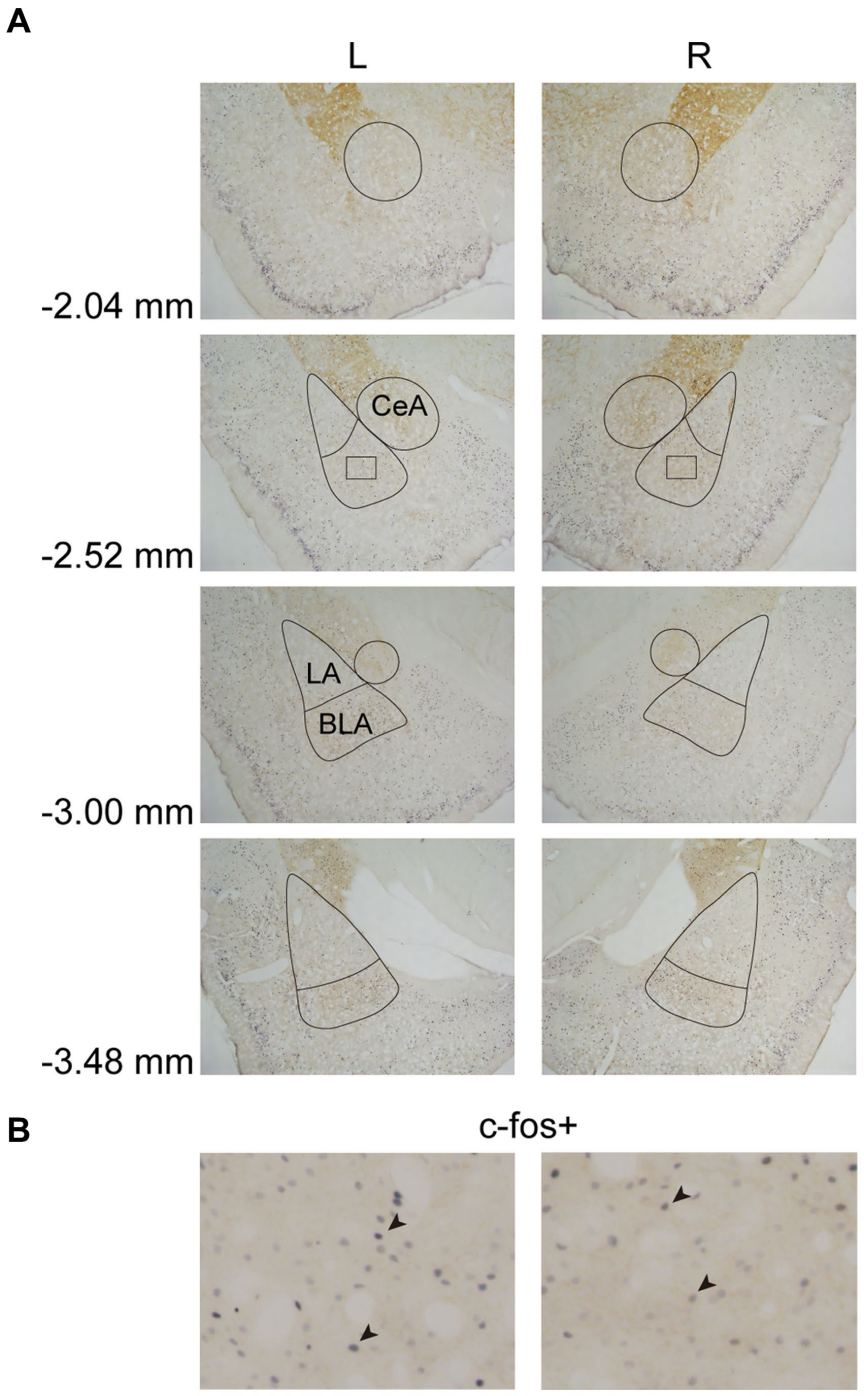
Example of c-fos + labelings in the amygdala. **(A)** The subregions of the amygdala were sampled at four anterior–posterior (AP) levels relative to bregma (−2.04, −2.52, −3.00, and −3.48 mm). The lines indicated the sampled area of the LA, the BLA, and the CeA. **(B)** Example of c-fos + neurons in the amygdala. The magnified images illustrate the rectangle areas shown in panel A. The c-fos + neurons were labeled in blue-black (four neurons were pointed with black arrowheads as examples). LA, lateral nucleus of the amygdala; BLA, basolateral nucleus of the amygdala; CeA, central nucleus of the amygdala.

**Figure 3:**
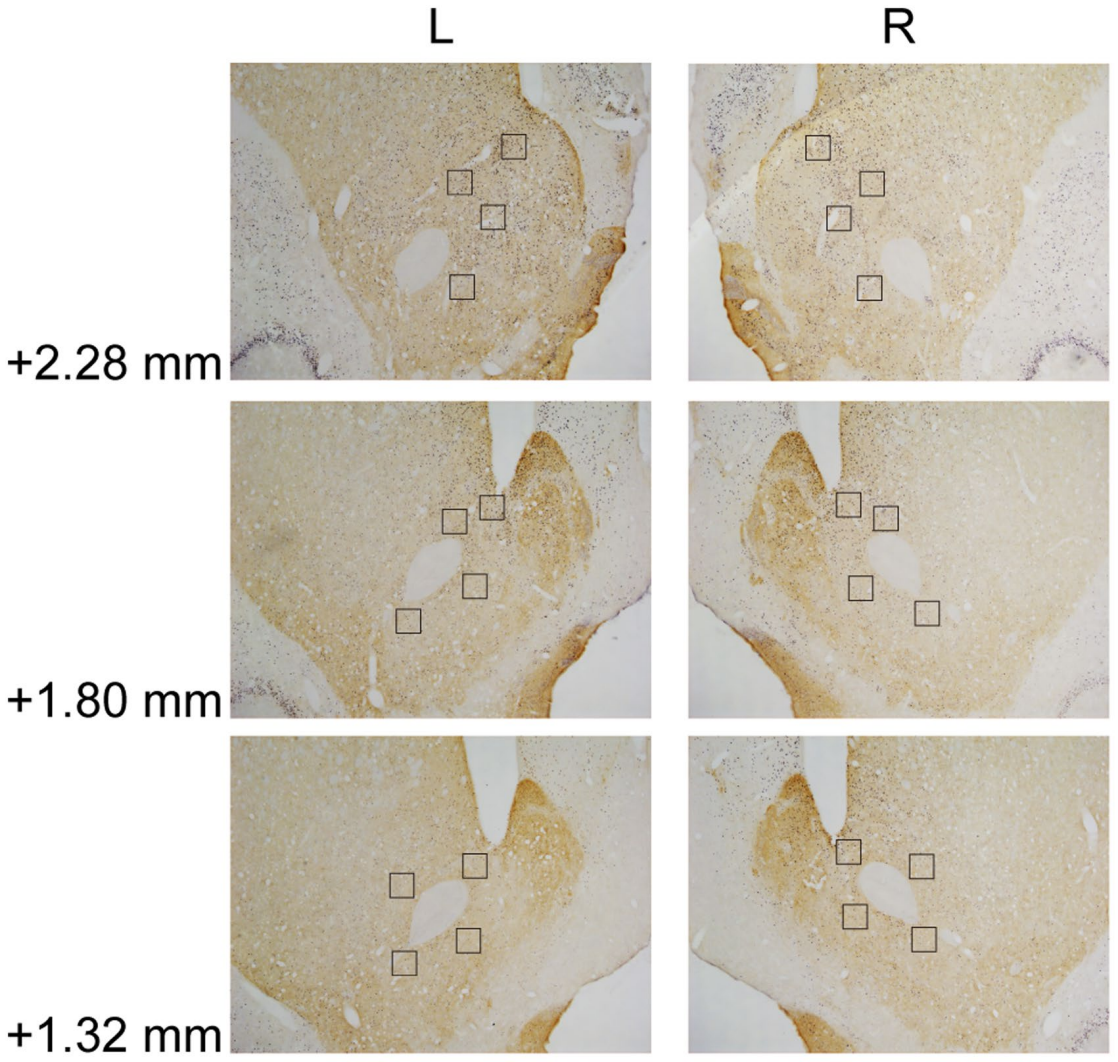
Example of c-fos + labelings in the NAcc. The NAcc was sampled at three anterior–posterior (AP) levels relative to bregma (+2.28, +1.80, and + 1.32 mm). The squares (200 × 200 μm^2^) represented the sampled area in each AP level. The c-fos + neurons were labeled in blue-black, as the same in Figure 2B. NAcc, nucleus accumbens core.

**Figure 4:**
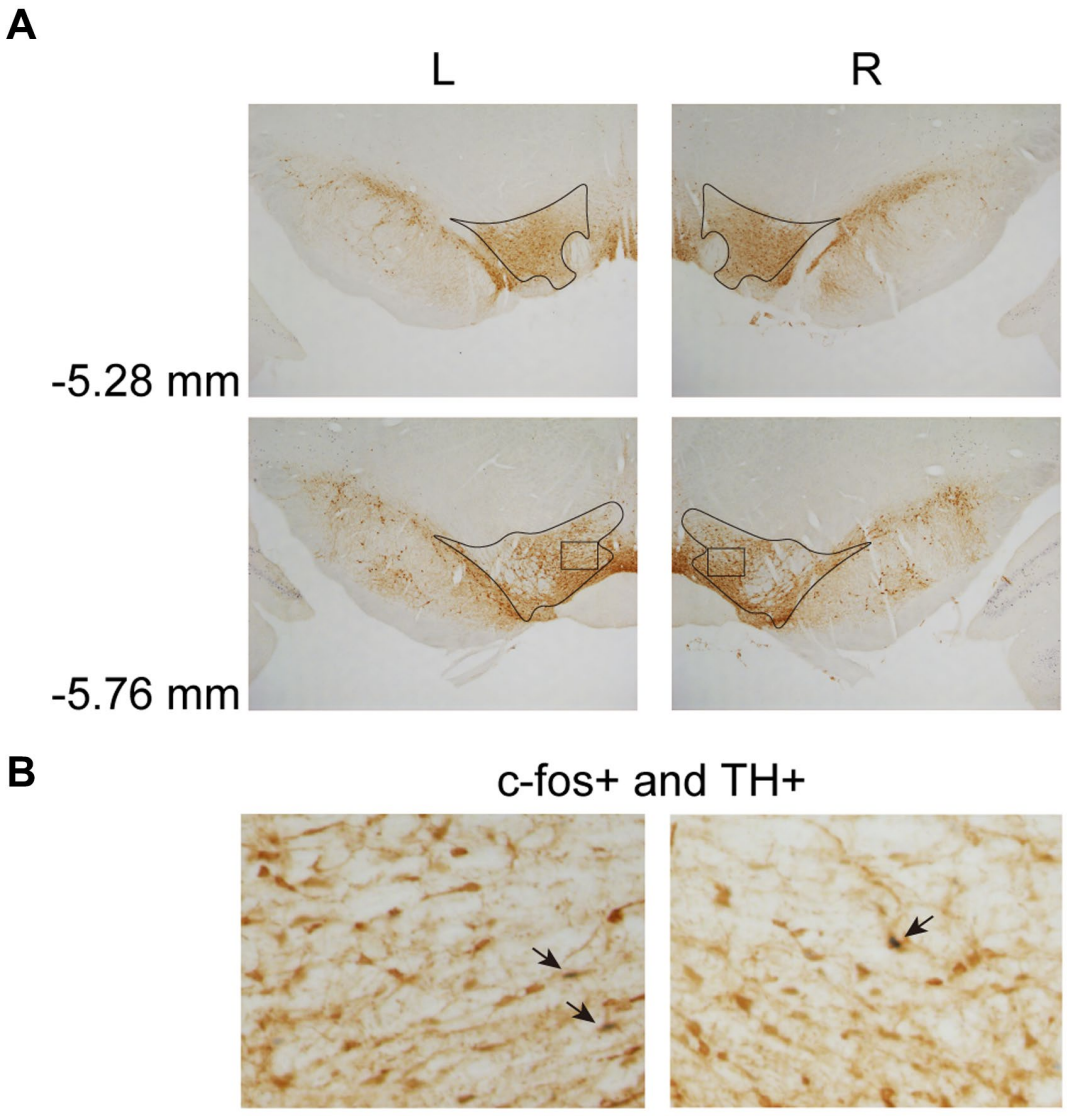
Example of c-fos + labelings of dopaminergic neurons in the VTA. **(A)** The VTA was sampled at two anterior–posterior (AP) levels relative to bregma (−5.28 and − 5.76 mm). **(B)** Example of labeled neurons in the VTA. The magnified images illustrate the rectangle areas shown in panel A. The c-fos+/TH+ neurons were identified as brown cell bodies with blue-black nuclei (three neurons were pointed with black arrowheads as examples). VTA, ventral tegmental area.

In Experiment 3, the images were taken with a digital camera on a microscope (Nikon ECLIPSE Ni-U). We manually counted the c-fos + neurons in the amygdala, the NAcc, and the VTA. The c-fos+/TH+ neurons were also counted in the VTA. The sampled amygdala regions were chosen at three levels of the LA/BLA [from bregma: (AP) −2.52, −3.00, and −3.48 mm] and the CeA [from bregma: (AP) −2.04, −2.52, and −3.00 mm] (Figure 2A). Examples of c-fos + neurons in sampled amygdala are presented in Figure 2B. The sampled NAcc regions were chosen at three levels [from bregma: (AP) +2.28, +1.80, and +1.32 mm]. The border of the NAcc was hard to define on brain slices, so the sampled areas were four 200 × 200 μm^2^ (40,000 μm^2^) squares surrounding the anterior commissure (Figure 3). The signals of c-fos + neurons in the NAcc are of the same quality as the ones shown in the amygdala (Figure 2B). The sampled VTA regions were chosen at two levels [from bregma: (AP) −5.28 and − 5.76 mm] (Figure 4A). Examples of c-fos+/TH+ neurons in sampled VTA are presented in Figure 4B. The sampled area of the respective subregions in the amygdala and the VTA were calculated with image analysis software (Toupview). The sampled area of the NAcc was the sum of sampled squares (480,000 μm^2^/side). The normalized values (count/μm^2^) were timed 10^6^ to yield cell density (count/nm^2^) for easier comprehension and analysis purposes.

### 2.9. Statistics

In Experiment 1, for cue-induced reinstatement test, lever pressing numbers from animals infused with saline were submitted to ANOVA with “Cannula” (BLA vs. CeA) as between-subject factor, and “Lever” (Active vs. Inactive lever) and “Session” (Last extinction vs. Reinstatement test) as within-subject factors. After confirming that there were no statistical differences in the “Cannula” main effect and related interactions, the animals were pooled into a combined saline group. The combined saline group was abbreviated as “SAL,” while the BLA and CeA activation groups were abbreviated as “BLA” and “CeA,” respectively. The lever pressing numbers were then submitted to ANOVA with “Group” (SAL vs. BLA vs. CeA) as between-subject factor, and “Lever” (Active vs. Inactive lever) and “Session” (Last extinction vs. Reinstatement test) as within-subject factors. For locomotor and free-feeding, the data from animals infused with saline were analyzed with the Student’s *t-*test. After confirming that there was no statistical difference between saline infusion in the BLA and the CeA, the animals were pooled into a combined saline group. The data were then submitted to ANOVA with “Group” (SAL vs. BLA vs. CeA) as between-subject factors. In Experiment 2, the data of EPM and FST were analyzed with a student’s t-test. In Experiment 3, for the cue-induced reinstatement test, the lever pressing numbers were submitted to ANOVA with “Group” (Ctrl vs. Stress) as between-subject factor, and “Lever” (Active vs. Inactive lever) and “Session” (Last extinction vs. Reinstatement test) as within-subject factors. The nosepoke counts were submitted to ANOVA with “Group” (Ctrl vs. Stress) as between-subject factor and “Session” (Last extinction vs. Reinstatement test) as within-subject factor. The nosepoke latency was analyzed with a student t-test. For cell counts, the cell density was submitted to ANOVA with “Group” (Ctrl vs. Stress) and “Reinstatement” (No Rein vs. Rein) as between-subject factors, and “Hemisphere” (Left vs. Right) as within-subject factor. Student–Newman–Keuls (S-N-K) *post hoc* was performed if significant *p*-values (*p* < 0.05) were obtained. All statistics were calculated using SPSS (IBM) and presented as mean ± SEM.

## 3. Results

### 3.1. Experiment 1: activation of the BLA or the CeA decreased cue-induced motivation toward food rewards in reinstatement test

**Figure 5:**
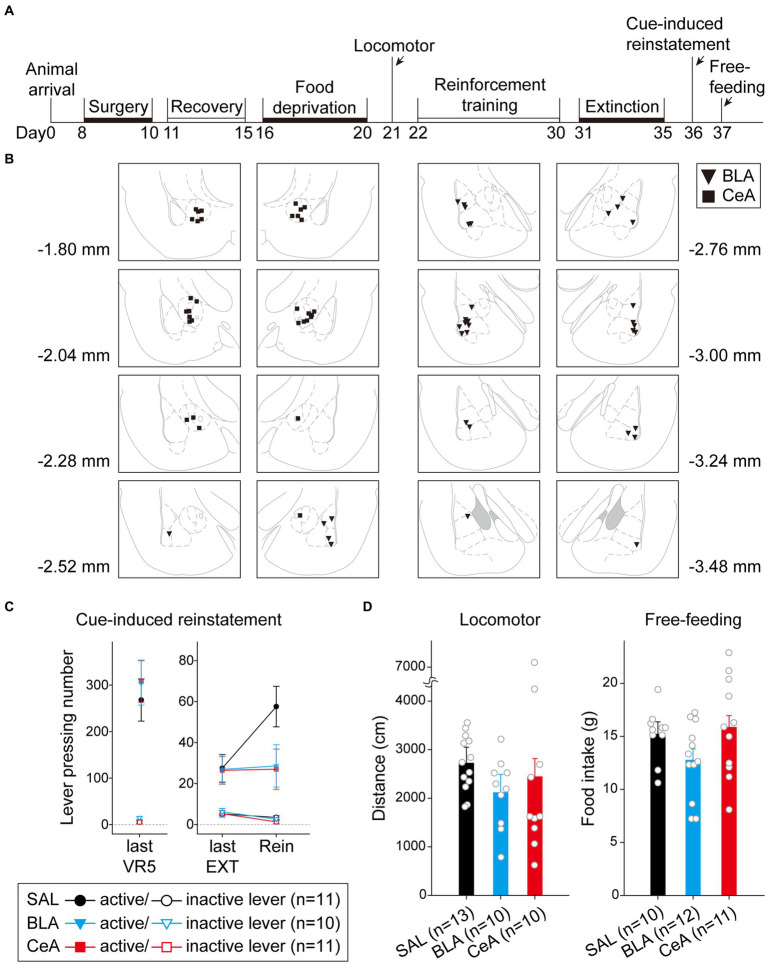
Activation of the BLA or the CeA decreased cue-induced motivation toward food rewards in the reinstatement test. **(A)** The experimental timeline in Experiment 1. The arrows indicate the time of drug administration. **(B)** The histology summary in Experiment 1. The numbers indicated anterior–posterior (AP) levels relative to bregma. **(C)** All groups of animals behaved equivalently on the last VR5 training day. Compared to the control group, activation of the BLA or the CeA decreased the reinstatement effect. **(D)** The locomotor activity and free-feeding test did not show differences among the three groups. All statistics were presented as mean ± SEM. BLA, basolateral nucleus of the amygdala; CeA, central nucleus of the amygdala; VR5, variable-ratio 5; EXT, extinction; Rein, Reinstatement test.

In this experiment, after recovering from surgery, the rats were food-deprived until the end of all behavioral tests. The rats were first tested with locomotor activity, followed by a cue-induced reinstatement test, and free-feeding test (Figure 5A). A total of 42 rats were used in this experiment. Nine subjects were excluded due to cannula misplacements, resulting in correct injector tip placements in the BLA (*n* = 17) or in the CeA (*n* = 16) for final analyses. The drug injection sites of subjects included for final analyses are summarized in Figure 5B. The counterbalance of drug administration is summarized in Table 1.

**Table 1:** Counterbalance of drug administration of Experiment 1.

Drug	Cannula placement		Total
	BLA	CeA	
S-N-S	1	1	2
S-N-N	2	2	4
S-S-N	2 + 1^a^	1	4
S-S-S	1	2	3
N-N-S	2	2	4
N-N-N	5	6	11
N-S-S	1	0	1
N-S-N	2	2	4
Total	17	16	33

Drug administration (S, Saline; N, NMDA) was presented in order of locomotor activity (Final grouping: SAL, *n* = 13; BLA *n* = 10; CeA, *n* = 10), cue-induced reinstatement test (Final grouping: SAL, *n* = 11; BLA, *n* = 10; CeA, *n* = 11), and free-feeding test (Final grouping: SAL, *n* = 10; BLA, *n* = 12; CeA, *n* = 11). BLA, basolateral nucleus of the amygdala; CeA, central nucleus of the amygdala. ^a^One subject was excluded from reinstatement test because of low lever pressing rate during reinforcement training.

For the animals infused with saline in cue-induced reinstatement test (Figure 5C), there were no statistical differences in the main effect of “Cannula” [*F*(1,9) = 1.29, *p* = 0.28], and the related two-way or three-way interactions [all three *F*(1, 9)s < 1]. Therefore, the animals were pooled into the SAL group. On the last VR5 training day, there was a significant main effect of “Lever” [*F*(1, 29) = 111.17, *p* < 0.001], but no statistical differences in the main effect of “Group” [*F*(2, 29) < 1] or interaction between “Lever” and “Group” [*F*(2, 29) < 1]. The results showed that the three groups of animals responded equivalently on the last VR5 training day and learned that responding on the active lever led to food rewards. From the last EXT training day to reinstatement test, there was a significant main effect of “Lever” [*F*(1, 29) = 47.17, *p* < 0.001], significant two-way interactions between “Lever” and “Session” [*F*(1, 29) = 7.95, *p* = 0.009] and between “Session” and “Group” [*F*(2, 29) = 3.75, *p* = 0.036], and significant three-way interaction among “Lever,” “Session,” and “Group” [*F*(2,29) = 3.38, *p* = 0.048]. The results indicated that the rats responded more to the active lever than to the inactive lever. *Post hoc* analyses revealed that only the SAL group demonstrated cue-induced reinstatement during the test, these rats showed an increase in the number of lever-pressing compared to the last EXT training session (active lever; *p* = 0.013). Together, activation of the BLA or the CeA demolished the cue-induced food-seeking behavior in the reinstatement test.

**Figure 6:**
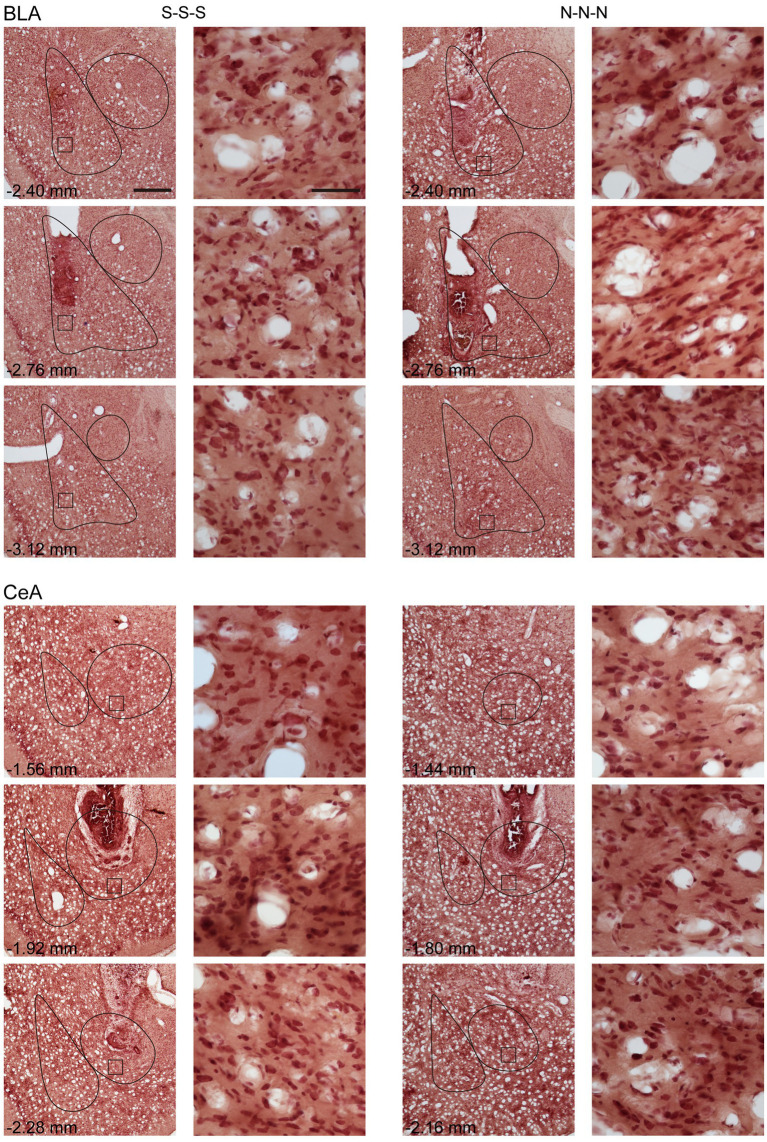
Example of neuronal morphology after three times of saline or NMDA infusion. For every subject, the brain sections were present with cannula implant location and 0.35 mm anterior or posterior relative to implant location. The magnified images illustrate the square areas shown in low-magnification images. Scale bar, 500 μm in low magnification images, 50 μm in high magnification images. BLA, basolateral nucleus of the amygdala; CeA, central nucleus of the amygdala; S, Saline, N, N-methyl-D-aspartate.

To rule out the possibility that activation of the amygdala may interfere with motor activity or lead to anhedonia in the animals, we tested locomotor activity and free-feeding test before reinforcement training and after the cue-induced reinstatement test, respectively (Figure 5D). For the animals infused with saline, there was no statistical difference between the animals with cannula implanted in the BLA or the CeA in locomotor activity [*t*(11) = 0.27, *p* = 0.79] or free-feeding test [*t*(8) = 0.47, *p* = 0.65]. Therefore, the animals were pooled into respective SAL groups. For locomotor activity, there was no statistical difference in the traveling distance among the three groups [*F*(2, 30) < 1]. For the free-feeding test, there was no statistical difference in consumed food pellets (g) among the three groups [*F*(2, 30) = 2.28, *p* = 0.12]. Together, activation of the BLA or the CeA did not change the locomotor activity of the animals or free food intake to the animals. Finally, since the animals received drug infusion three times, we checked the nissl-stained sections to rule out excitotoxic lesions. The implantation caused physical tissue damage surrounding the cannula, but the adjacent neurons remained unaffected after pharmacological activation. There were no obvious signs of excitotoxic lesions, including loss of neurons or tissue, proliferation of microglial cells, or pyknotic nuclei (Hembrook and Mair, 2011). The neurons in the CeA are smaller because this nucleus mostly consists of gamma-aminobutyric acid (GABA) neurons, which are smaller than pyramidal neurons (McDonald, 1982a,b; McDonald and Mascagni, 2001). The exemplary nissl-stained sections and magnified images of subjects infused with NMDA or saline for all three behavioral tests are presented in Figure 6.

### 3.2. Experiment 2: single prolonged footshocks-induced acute stress did not affect anxiety-and despair-like behavior

**Figure 7:**
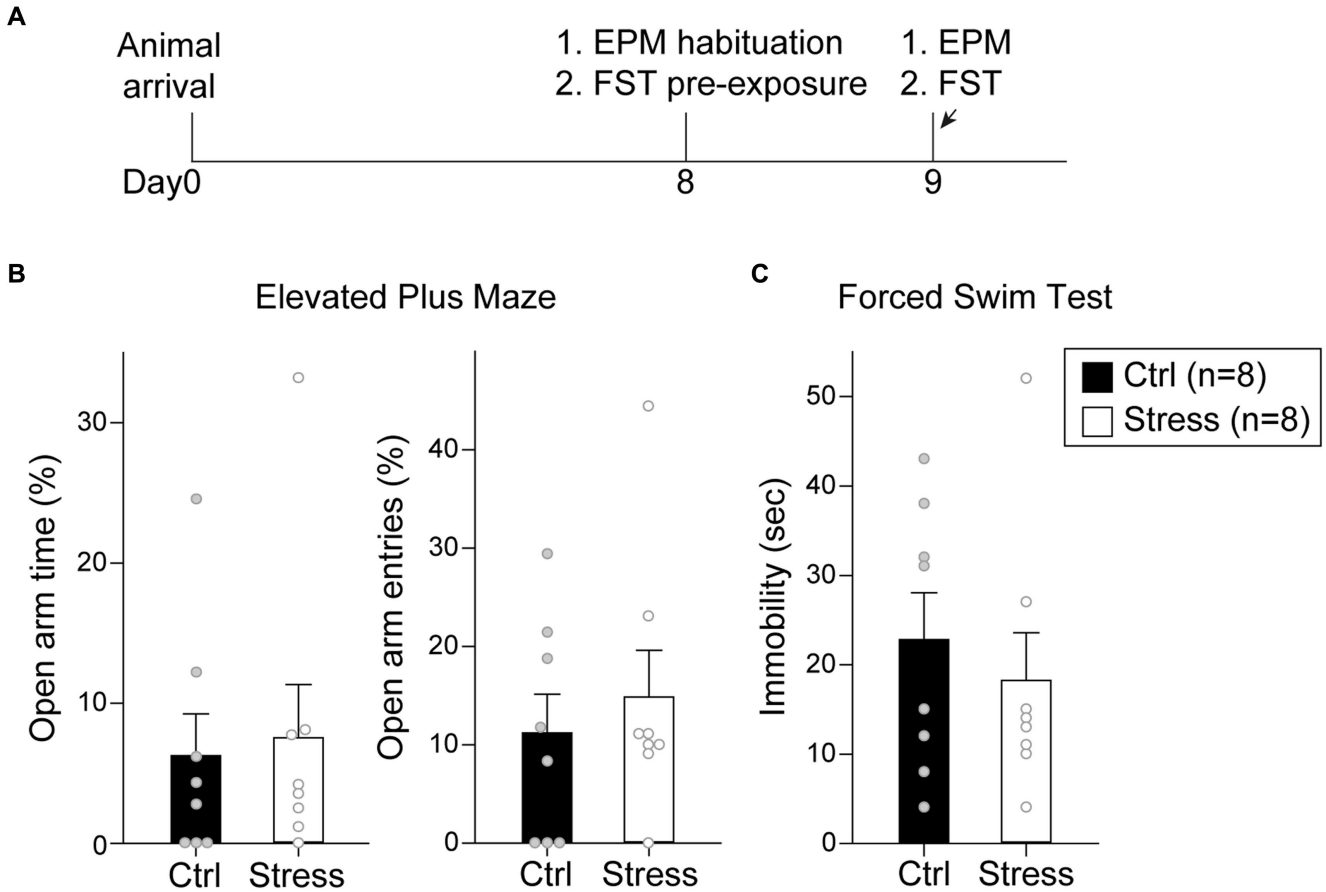
Footshock-induced acute stress did not affect anxiety-and despair-like behaviors. **(A)** The experimental timeline in Experiment 2. The arrows indicate the time of a single prolonged footshock-induced acute stress. There was no statistical difference between groups in **(B)** percentage of time spent in open arms and entries into open arms in EPM and **(C)** time spent in immobility in FST. All statistics were presented as mean ± SEM. EPM, elevated plus maze; FST, forced swim test.

In this experiment, the rats were tested with EPM and FST after a single prolonged footshock-induced stress session (Figure 7A). A total of 16 rats were used in this experiment, with groups assigned as “Ctrl” (*n* = 8) and “Stress” (*n* = 8). For EPM, there was no statistical difference in the percentage of time spent in the open arm [*t*(14) = 0.26, *p* = 0.80] or the percentage of entries into the open arm [*t*(14) = 0.59, *p* = 0.57] (Figure 7B). For FST, there was no statistical difference in the time spent in immobility [*t*(14) = 0.62, *p* = 0.55] (Figure 7C). The results suggested that single prolonged footshock-induced stress did not interfere with the anxiety-and despair-like behaviors of the animals.

### 3.3. Experiment 3: single prolonged footshocks-induced acute stress did not affect cue-induced motivation toward food rewards in the reinstatement test

**Figure 8:**
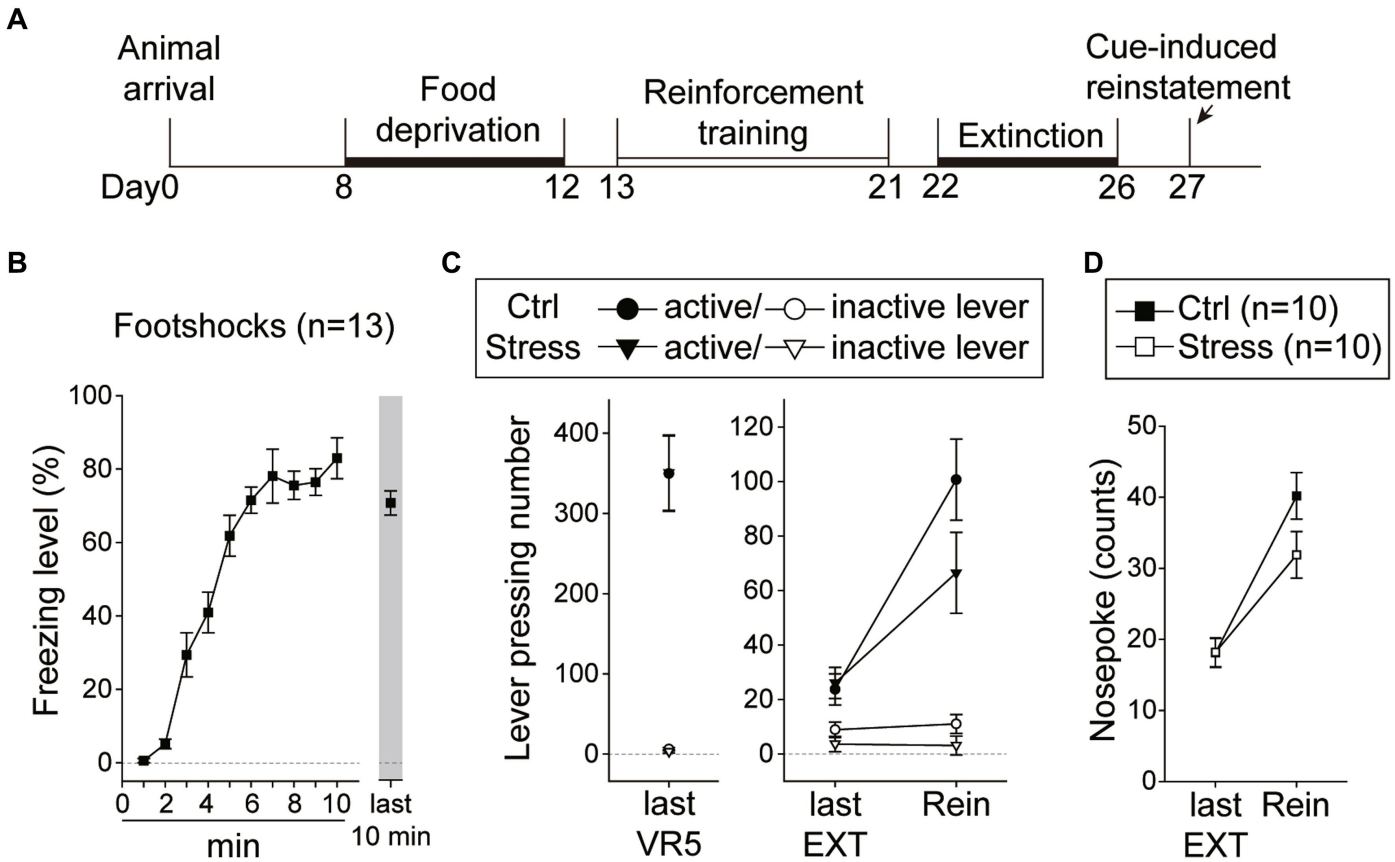
Footshock-induced acute stress did not interfere with cue-induced motivation toward food rewards in the reinstatement test. **(A)** The experimental timeline in Experiment 3. The arrow indicates the time of a single prolonged footshock-induced acute stress. **(B)** The freezing level during the first 10 min and the last 10 min of the total 2 h session. **(C)** The two groups of animals that underwent the reinstatement procedure behaved equivalently on the last VR5 training day. The footshock-induced acute stress did not decrease the motivation of food-seeking. Both the Stress and Ctrl group of the animals showed reinstatement to cues. **(D)** The footshock-induced acute stress did not decrease nosepoke counts from the last extinction session to the reinstatement test in both groups. All statistics were presented as mean ± SEM. VR5, variable-ratio 5; EXT, extinction; Rein, Reinstatement test.

In this experiment, the rats underwent reinforcement training and extinction, and were food-deprived until the end of all behavioral tests (Figure 8A). Immediately before the cue-induced reinstatement test, the rats underwent a single prolonged footshock-induced stress session. A total of 32 rats were used in this experiment, with group assignment as follows: “Ctrl-Rein” (*n* = 10), “Stress-Rein” (*n* = 10), “Ctrl-No Rein” (*n* = 6), and “Stress-No Rein” (*n* = 6). For the single prolonged footshocks session (“Stress,” *n* = 16), the video recordings of three animals were lost due to a program crash, and therefore the freezing level was analyzed based on the remaining 13 subjects. For these rats that received footshocks (Figure 8B), they showed a rapid increase in freezing level during the first 10 min, which remained at a high level toward the end of the 2 h session. The “No Rein” animals (“Ctrl-No Rein” and “Stress-No Rein”) did not undergo the reinstatement test after the stress session. These two groups served as the controls to examine how single prolonged footshock-induced stress may have affected cellular activities.

For the two groups of animals (“Ctrl-Rein” and “Stress-Rein”) that underwent the reinstatement test (Figure 8C), on the last VR5 training day, there was a significant main effect of “Lever” [*F*(1, 18) = 108.39, *p* < 0.001], but no statistical differences in the main effect of “Group” [*F*(1, 18) < 1] or interaction between “Lever” and “Group” [*F*(1, 18) < 1]. The results showed that these animals responded equivalently on the last VR5 training day and learned that responding on the active lever led to food rewards. From the last EXT training day to the reinstatement test, there were significant main effects of “Lever” [*F*(1, 18) = 76.73, *p* < 0.001] and “Session” [*F*(1, 18) = 30.19, *p* < 0.001], and a significant two-way interaction between “Lever” and “Session” [*F*(1, 18) = 41.71, *p* < 0.001]. However, there were only a marginal effect of two-way interaction between “Session” and “Group” [*F*(1, 18) = 3.28, *p* = 0.087] and a marginal three-way interaction among “Lever,” “Session,” and “Group” [*F*(1, 18) = 3.60, *p* = 0.074]. Because of the marginal effects, we further analyzed nosepoke as another index of motivation (Figure 8D). From the last EXT training day to the reinstatement test, there was a significant main effect of “Session” [*F*(1, 18) = 88.80, *p* < 0.001], and a significant two-way interaction between “Session” and “Group” [*F*(1, 18) = 4.90, *p* = 0.04]. However, *post hoc* analyses revealed that both “Ctrl” and “Stress” groups showed an increase in nosepoke counts compared to their respective last EXT training session (*p*s < 0.001) and there was only a marginal difference between the two groups on the reinstatement test day (*p* = 0.09). The omission rates were very low and there was no difference in nosepoke latency between the two groups [*t*(18) = 1.20, *p* = 0.25] with 2.81 ± 0.35 s in the “Ctrl” group and 3.75 ± 0.71 s in the “Stress” group. These results suggested that both groups showed a reinstatement effect and footshocks-induced acute stress did not decrease the motivation in cue-induced food-seeking behavior.

**Figure 9:**
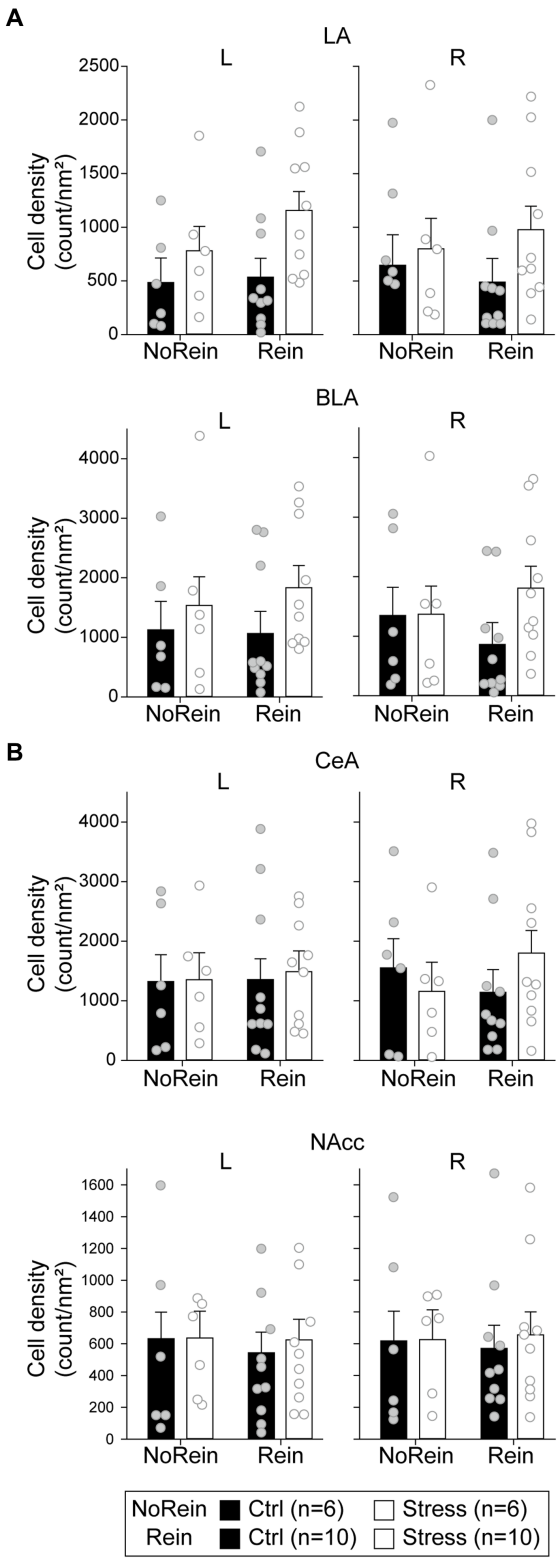
The cell density in (count/nm^2^) in **(A)** the amygdala (the LA, the BLA, and the CeA) and **(B)** the NAcc. All statistics were presented as mean ± SEM. LA, lateral nucleus of the amygdala; BLA, basolateral nucleus of the amygdala; CeA, central nucleus of the amygdala; NAcc, nucleus accumbens core.

To analyze the cellular activities during the reinstatement test, we sampled the c-fos + neurons in the amygdala, the NAcc, and the VTA. In the LA (Figure 9A, upper panels), there were only a marginal main effect of “Group” [*F*(1, 28) = 3.05, *p* = 0.092] and a marginal two-way interaction between “Hemisphere” and “Reinstatement” [*F*(1, 28) = 3.11, *p* = 0.089]. In the BLA (Figure 9A, middle panels), there was only a marginal three-way interaction among “Hemisphere,” “Group,” and “Reinstatement” [*F*(1, 28) = 3.41, *p* = 0.076]. In the CeA (Figure 9A, lower panels), there was a significant three-way interaction among “Hemisphere,” “Group” and “Reinstatement” [*F*(1,28) = 4.47, *p* = 0.043]. However, *post hoc* analyses revealed that there was no statistical difference between the “Ctrl” and “Stress” groups in either hemisphere regardless the animals underwent reinstatement or not. Together, we did not find evidence that acute stress and/or reinstatement tests increased the cellular activities in the amygdala.

In the NAcc (Figure 9B), the cellular activities did not show any statistical difference among the groups. In the VTA, the cell densities of c-fos + or c-fos+/TH+ neurons were very low. Before normalizing to the sampled VTA area, the mean c-fos + cell count is 15.03 ± 3.79 (range 0–100) in the left VTA and 18.09 ± 4.62 (range 0–125) in the right VTA. The mean c-fos+/TH+ cell count is 5.38 ± 1.24 (range 0–30) in the left VTA and 6.69 ± 1.50 (range 0–32) in the right VTA. Therefore, we did not further analyze the data considering the results were likely biased. The minimum, maximum, and mean cell density in respective brain regions were summarized in Table 2.

**Table 2:** Cell density (count/nm^2^) of c-fos + or c-fos+/TH+ neurons in the amygdala, the NAcc, and the VTA.

	LA		BLA		CeA	
	L	R	L	R	L	R
Min	16.22	100.01	76.91	68.08	199.63	57.85
Max	2124.39	2325.02	4383.71	4042.12	3889.48	3979.41
Mean	766.31	730.14	1405.08	1343.63	1391.87	1429.11
	NAcc		VTA^a^		VTA^b^	
	L	R	L	R	L	R
Min	112.50	125.00	0.00	0.00	0.00	0.00
Max	1652.08	1672.92	585.30	646.88	175.59	165.60
Mean	603.06	616.47	96.75	93.90	34.73	35.03

LA, lateral nucleus of the amygdala; BLA, basolateral nucleus of the amygdala; CeA, central nucleus of the amygdala; NAcc, nucleus accumbens core; VTA, ventral tegmental area; TH, tyrosine hydroxylase; L, left; R, right. ^a^c-fos + only neurons in the VTA. ^b^c-fos+/TH+ neurons in the VTA.

## 4. Discussion

In this study, we aimed to investigate how amygdala activation or physical acute stress sessions would affect cue-induced motivation in rats. The behavioral results showed that pharmacological activation of the BLA or the CeA decreased cue-induced motivation toward foods. However, single prolonged footshock-induced stress did not immediately affect anxiety-, despair-like behavior, or motivation toward food. The cellular activities assessed in the amygdala subnuclei or the NAcc also showed no difference among hemispheres and groups after acute stress and/or reinstatement test. Together, only intra-cranial pharmacological activation of the amygdala led to the decrease in appetitive motivated behavior.

In Experiment 1, pharmacological activation of the BLA or the CeA decreased the cue-induced motivation of the animals. This result further supported the idea that down-regulation of the DA population activity following BLA activation would suppress the reward system (Belujon and Grace, 2015, 2017). Moreover, activation of either the BLA or the CeA led to similar behavioral outcomes, which was consistent with the concept of serial information flow from the BLA to the CeA that guided many motivated behaviors (Beyeler et al., 2016; Kim et al., 2017). However, we could not rule out the possibility that the BLA and the CeA were under the regulation of a common upstream brain region. Indeed, some previous research has suggested that the BLA and the CeA mediated emotional processing parallelly (Balleine and Killcross, 2006; Moscarello and Ledoux, 2013). Since the BLA and the CeA were adjacent to each other, the drug was delivered at the rate of 0.2 μL/min for 2 min 30 s, which was slower than the procedure of previous studies (0.5 μL/min for 1 min) (Rezayof et al., 2007; Solati and Hajikhani, 2010). However, we cannot rule out the possibility that the drug spreads beyond the targeted region. A better solution is to use chemogenetics in future studies so that the precise expression of the receptors can be confirmed.

The amygdala is not only involved in the regulation of negative emotions but also positive emotions, such as reward processing (Baxter and Murray, 2002; Murray, 2007). The amygdala activity is important during the encoding and retrieval phase of stimulus-reinforcement association (Schoenbaum et al., 1998, 1999; Malvaez et al., 2019; Crouse et al., 2020). In the study of McLaughlin and Floresco, inhibition of caudal BLA enhanced reinstatement in food-seeking (McLaughlin and Floresco, 2007). Our results are in line with this report and showed that activation of the BLA or the CeA abolished cue-induced food-seeking after extinction. Beyond the natural reward, such as sucrose pellets or solution, most of the studies regarding the role of the amygdala in reinstatement focused on drug-seeking (Sharp, 2017). For example, lesion or inhibition of the amygdala attenuated reinstatement of drug-seeking (Kantak et al., 2002; McLaughlin and See, 2003; See et al., 2003; Fuchs et al., 2004a). Inhibition of the BLA to NAcc pathway abolished the reinstatement of drug-seeking as well (Stefanik and Kalivas, 2013). Based on these contradictory results, the underlying mechanisms of reinstatement of drug-and food-seeking are likely different and need further investigation.

In Experiment 2, single prolonged footshock-induced acute stress did not increase the anxiety level in EPM or the despair level in FST. In Experiment 3, we further analyzed the freezing behavior during the acute stress session and confirmed that the animals did show a high level of fear toward the end of the session. However, in our cue-induced reinstatement, there was only a marginal effect of a three-way interaction among “Lever,” “Session,” and “Group” (*p* = 0.074) in the lever pressing number. Although there was a significant two-way interaction between “Session” and “Group” in nosepoke counts, the count difference between the “Ctrl” and “Stress” groups on the reinstatement test day was only marginal (*p* = 0.09), suggesting that the animals only showed a trend of decrease in the motivation of food-seeking after acute stress. Considering that the acute stress did not increase the negative emotions at the time point of our assessment, it is possible that the stress effect was weak and therefore resulted in a marginal effect on the reinstatement test. It has been suggested that stress is a risk factor to induce reinstatement of drug-seeking (Shaham et al., 2003; Shalev et al., 2010; Mantsch et al., 2015). For stress-induced reinstatement in food-seeking, the findings remain inconclusive. For example, studies from Shaham’s group demonstrated that yohimbine, which produces a stress-like response in animals, induced reinstatement in food-seeking (Ghitza et al., 2005; Nair et al., 2010; Lê et al., 2011). However, intermittent footshock-induced stress promoted (Chen et al., 2014) or had no effect (Ahmed and Koob, 1997; Buczek et al., 1999) on reinstatement in food-seeking, whereas a recent study revealed that acute footshock-induced stress decreased appetitive learning and motivated behavior (Derman and Matthew Lattal, 2023). In human studies, after acute stress such as threat-of-shock or cold condition (Bogdan and Pizzagalli, 2006; Morris and Rottenberg, 2015; Burani et al., 2021), or in post-traumatic stress disorder (PTSD) patients (Hopper et al., 2008; Nawijn et al., 2015), these subjects displayed dysfunction of reward processing.

There are some operational factors that have led to the limitations of this study. First, the food deprivation process itself increased plasma corticosterone (CORT) levels (Guarnieri et al., 2012; Lenglos et al., 2013) or induced behavioral change, such as an anxiolytic effect in EPM of the animals (Genn et al., 2003; Dietze et al., 2016). However, food restriction is an inevitable step in cue-induced food-seeking tasks. Second, the control groups (“Ctrl,” non-stressed controls and “No Rein,” no reinstatement controls) were left in their home cages while their counterparts underwent corresponding behavioral procedures. In our experimental design, the single prolonged footshock-induced stress served as a distinct experience. This design paralleled other stress protocols using restraint (Kovács et al., 2018; Sousa et al., 2018) or chronic unpredictable mild stress (Nollet et al., 2013; Wiborg, 2013; Chang and Grace, 2014) that the control animals usually were left in their home cages, undisturbed. However, we acknowledged that placing the “Ctrl” animals into fear-conditioning cubicles without footshocks could have ruled out the environmental variables. For the “NoRein” controls, these animals remained in their home cages to examine the cellular activities due to stress *per se*. Similarly, we acknowledged that having another control group placed in the operant chamber without the presentation of food-related CS could have ruled out environmental variables, and also their lever pressing numbers could have served as a baseline for behavioral comparison.

The single prolonged stress session in this study was adapted from Arakawa’s study (Arakawa et al., 2014). In their study, female Sprague–Dawley rats were used as their subjects, and they demonstrated that the plasma CORT level was significantly increased immediately after footshocks. It has been suggested that females have higher activities of the HPA axis after acute stress and exhibit distinct fear responses from male subjects (Goel et al., 2014; Bangasser and Wicks, 2017; Heck and Handa, 2018). However, as only male Lone-Evans rats were used as our subjects and we did not measure the CORT level of our “Ctrl” and “Stress” animals, there was no direct physiological evidence of stress measurement. Next, the type of stressor is also likely to influence stress response. In studies using footshock as the stressor, the intensity of footshock ranged from 0.5–2.0 mA, and the duration of session time lasted from under 30 min (Haj-Mirzaian et al., 2014; Ness et al., 2022), to 1 h (Shinba et al., 2010), or up to 2 h (Blandino et al., 2006). Therefore, it is hard to directly compare the intensity of stress among all these protocols. To study the animal model of PTSD, the single prolonged stress procedure is usually a combination of several stressors, such as footshock, forced swim, and anesthetization (Knox et al., 2012; Eagle et al., 2013; Souza et al., 2017; Lisieski et al., 2018). It is possible that using only the footshock as in our stress protocol was not intense enough to alter behaviors of the animals. Finally, we noticed that the intermittent footshock of earlier studies was performed in the same operant chamber as the reinstatement test, while in our experiments, the footshocks were performed in other chambers with a different context setting. It is likely that the shift in context between the acute stress session and the reinstatement test was regarded as a safety signal in some of the animals, and therefore, these animals displayed reinstatement behaviors. Indeed, earlier studies have suggested that the context and mental state during training contributed to the renewal phenomenon in operant behaviors (Bouton et al., 2012; Sweeney and Shahan, 2015). Moreover, one study showed that the food-seeking behavior was reinstated after stress only when the stress was also introduced during reinforcement training sessions (Schepers and Bouton, 2019), implying that the reinstatement might be state-dependent.

Since previous studies showed that the amygdala was immediately activated under acute stress (Cullinan et al., 1995; Reznikov et al., 2007, 2008) and also to parallel the experimental design of Experiment 1, we conducted the behavioral tests and sampled the cellular activities with c-fos expression immediately after the acute stress in Experiment 3. Similar to the behavioral results, there were only marginal effects of the cellular activities. In the LA, the “Stress” animals showed a trend of higher cellular activities than “Ctrl” animals. At the level of the BLA and the CeA, the “Stress” animals only showed a trend of higher cellular activities in the right hemisphere when they underwent reinstatement tests. In general, the “Stress” animals tended to have higher cellular activities in the amygdala compared to “Ctrl” ones if they underwent reinstatement tests. One possibility is that the acute stress procedure prepared the animals to be alert, which is similar to the “pre-encounter defensive behavior” as stated in the predatory imminence theory. Under this scenario, the “Stress” animals showed lower motivated behavior during the reinstatement test, accompanied by higher cellular activities in the amygdala. However, earlier studies assessed behavioral performance after acute stress with different temporal delays, from immediately (Masood et al., 2004; Olonode et al., 2018), 1 day (Mitra et al., 2005; Suvrathan et al., 2010), or even 10 days (Mitra et al., 2005; Kedia and Chattarji, 2014) afterward. This temporal delay after the stress procedure could be another factor leading to our marginal effects at both the behavioral and cellular levels.

Other than the amygdala, we also assessed the cellular activities of the reward system, including the VTA and the NAcc. However, the cell densities of c-fos + DA and non-DA neurons in the VTA were very low, and the cellular activities in the NAcc did not show statistical differences among groups. Studies suggested that the DA mesolimbic transmission initially increased after acute stress, but subsequently decreased (Puglisi-Allegra et al., 1991; Valenti et al., 2011; Chang and Grace, 2013). Moreover, our previous research showed that BLA activation decreased VTA population activity (Chang and Grace, 2014; Lai and Chang, 2019). Therefore, the alteration of activities in the VTA may depend on several variables. The VTA DA system is important in reward processing, especially stimulus–reward learning and the motivation to obtain reward (Arias-Carrián et al., 2010; Ranaldi, 2014; Salamone et al., 2016). Most of the research focused on drug-seeking behavior, for example, blocking glutamate receptors (McFarland and Kalivas, 2001; Sun et al., 2005; Mahler et al., 2013) or orexin receptors (James et al., 2011; Mahler et al., 2013) in the VTA reduced reinstatement of cocaine-seeking in rats. It has been reported that blocking glutamate receptors in the VTA did not affect reinstatement of food-seeking (Sun et al., 2005), although “food-induced” reinstatement was used in this study. In the VTA, a large proportion of the neurons are DA neurons, with the rest non-DA neurons mostly consisting of GABAergic neurons (Nair-Roberts et al., 2008). The VTA DA neurons project to the medial prefrontal cortex (mPFC), the amygdala, the lateral habenula (LHb), and robustly to the NAc (Yetnikoff et al., 2014; Beier et al., 2015; Morales and Margolis, 2017). The VTA GABAergic neurons exert local inhibition on DA neurons or other VTA GABAergic neurons, as well as long-range inhibition to other brain regions, such as the mPFC, the LHb, and the NAc (Creed et al., 2014; Yetnikoff et al., 2014; Morales and Margolis, 2017; Bouarab et al., 2019). The DA and non-DA long-range projections modulated reward processing and aversive behaviors (Morales and Margolis, 2017; Bouarab et al., 2019). However, previous studies only suggested the importance of the VTA DA neuron in the reinstatement of cocaine-seeking (Tian et al., 2022). Thus, the cell-type specific contribution of the VTA neurons on the reinstatement of food-seeking remains unknown. In the NAc, earlier studies have demonstrated that DA transmission is important for reinforcement-related behaviors, especially in initiating and maintaining instrumental behaviors (Salamone and Correa, 2002; Salamone et al., 2003, 2005). For example, inactivation of the NAcc with GABA agonist reduced food-seeking (Floresco et al., 2008; Lin and Pratt, 2014) and cocaine-seeking (Fuchs et al., 2004b) behavior during the reinstatement test. In our results, footshock-induced stress only resulted in a trend of decrease in cue-induced motivation of the animals. The fact that the stressed animals did show an effect of reinstatement might have explained the lack of difference in cellular activities in the NAcc assessed through c-fos expression.

Our results showed that pharmacological activation of the amygdala during the reinstatement test significantly suppressed the cue-induced motivation of the animals (Experiment 1). The single prolonged footshock-induced acute stress did not affect anxiety-, despair-like behaviors (Experiment 2), or motivation of food-seeking (Experiment 3). Therefore, activation of the amygdala *per se* did not fully capture the state of the animals being under acute stress. Indeed, multiple systems may be engaged under acute stress, including the HPA axis (Johnson et al., 1992; Kudielka and Kirschbaum, 2004) and the sympathetic division of ANS (McCorry, 2007). Some animal studies suggested that glucocorticoids released under stress enhanced response to rewards (Piazza and Le Moal, 1997; Marinelli and Piazza, 2002; Lemaire et al., 2005), while in human subjects with heightened cortisol levels after acute stress led to deficits in reward processing (Berghorst et al., 2013) and administration of cortisol to human subjects decreased the neuronal activities relative to reward (Montoya et al., 2014; Kinner et al., 2016). The glucocorticoid and noradrenergic activities combined also reduced goal-directed actions in humans (Schwabe et al., 2012). Together, our results further supported the idea that stress response is a complicated coping process that engages many systems beyond the ones mediated through the central nervous systems via the amygdala. The mechanism of how stress affects motivation demands future research.

## Data availability statement

The original contributions presented in the study are included in the article/supplementary material, further inquiries can be directed to the corresponding author.

## Ethics statement

The animal study was approved by the Institutional Animal Care and Use Committees (IACUC) of both National Tsing Hua University and National Yang Ming Chiao Tung University. The study was conducted in accordance with the local legislation and institutional requirements.

## Author contributions

C-WL: conceptualization, methodology, formal analysis, investigation, writing-original draft, visualization, project administration. C-hC: conceptualization, methodology, validation, and resources, writing-review and editing, visualization, supervision, and funding acquisition. All authors contributed to the article and approved the submitted version.

## Funding

This work was supported by the National Science and Technology Council (Taiwan) (Grant no. 111-2628-B-007-008-) to C-hC and the Brain Research Center under the Higher Education Sprout Project, co-funded by the Ministry of Education and the National Science and Technology Council (Taiwan).

## Conflict of interest

The authors declare that the research was conducted in the absence of any commercial or financial relationships that could be construed as a potential conflict of interest.

## Publisher’s note

All claims expressed in this article are solely those of the authors and do not necessarily represent those of their affiliated organizations, or those of the publisher, the editors and the reviewers. Any product that may be evaluated in this article, or claim that may be made by its manufacturer, is not guaranteed or endorsed by the publisher.
